# Lack of Cross-Scale Linkages Reduces Robustness of Community-Based Fisheries Management

**DOI:** 10.1371/journal.pone.0006253

**Published:** 2009-07-16

**Authors:** Richard Cudney-Bueno, Xavier Basurto

**Affiliations:** 1 School of Natural Resources, University of Arizona, Tucson, Arizona, United States of America; 2 Institute of Marine Sciences, Long Marine Laboratories, University of California Santa Cruz, Santa Cruz, California, United States of America; 3 Centro Intercultural de Estudios de Desiertos y Océanos, Puerto Peñasco, Sonora, México; 4 Workshop in Political Theory and Policy Analysis, Indiana University, Bloomington, Indiana, United States of America; 5 Duke Marine Lab, Nicholas School of the Environment, Duke University, Beaufort, North Carolina, United States of America; Duke University, United States of America

## Abstract

Community-based management and the establishment of marine reserves have been advocated worldwide as means to overcome overexploitation of fisheries. Yet, researchers and managers are divided regarding the effectiveness of these measures. The “tragedy of the commons” model is often accepted as a universal paradigm, which assumes that unless managed by the State or privatized, common-pool resources are inevitably overexploited due to conflicts between the self-interest of individuals and the goals of a group as a whole. Under this paradigm, the emergence and maintenance of effective community-based efforts that include cooperative risky decisions as the establishment of marine reserves could not occur. In this paper, we question these assumptions and show that outcomes of commons dilemmas can be complex and scale-dependent. We studied the evolution and effectiveness of a community-based management effort to establish, monitor, and enforce a marine reserve network in the Gulf of California, Mexico. Our findings build on social and ecological research before (1997–2001), during (2002) and after (2003–2004) the establishment of marine reserves, which included participant observation in >100 fishing trips and meetings, interviews, as well as fishery dependent and independent monitoring. We found that locally crafted and enforced harvesting rules led to a rapid increase in resource abundance. Nevertheless, news about this increase spread quickly at a regional scale, resulting in poaching from outsiders and a subsequent rapid cascading effect on fishing resources and locally-designed rule compliance. We show that cooperation for management of common-pool fisheries, in which marine reserves form a core component of the system, can emerge, evolve rapidly, and be effective at a local scale even in recently organized fisheries. Stakeholder participation in monitoring, where there is a rapid feedback of the systems response, can play a key role in reinforcing cooperation. However, without cross-scale linkages with higher levels of governance, increase of local fishery stocks may attract outsiders who, if not restricted, will overharvest and threaten local governance. Fishers and fishing communities require incentives to maintain their management efforts. Rewarding local effective management with formal cross-scale governance recognition and support can generate these incentives.

## Introduction

Coastal fishing communities are increasingly exposed to global market pressures, making them more vulnerable to “roving bandits” who can deplete local fishing stocks and move on to other areas to do the same [1], seriously threatening ecosystems and the people who depend on them to survive [2], [3], especially those located in developing countries [4]. To overcome the threat of roving bandits and overexploitation of fisheries, international financial organizations and some national governments are investing huge sums to foster the establishment of marine reserves and community-based management (CBM) [5], [6].

The research community, however, is divided regarding the potential effectiveness of CBM for developing sustainable fisheries [7]. The capabilities of managing coastal fisheries locally, although well documented [8]–[11] have often been ignored or criticized, viewed as relics that are irrelevant to contemporary situations [12], [7]. Indeed, local fisheries are rapidly appearing and—just as rapidly—disappearing in response to emerging global markets and overfished stocks [1], leaving little time to develop effective customary management practices with which to avoid local overexploitation. Many fishery officials and scholars still accept “the tragedy of the commons” model [13] that assumes that due to conflicts between the self-interest of members of a group and the goals of a group as a whole, common-pool resources need to be managed by the State or privatized to avoid overexploitation. Under this paradigm, the emergence and maintenance of effective community-based efforts that include costly and risky decisions as the establishment of marine reserves would not occur, particularly in recently organized fisheries.

In this study, we question these assumptions and show that the realities of commons dilemmas can be complex and scale dependent. Recently organized fisheries have the potential to develop effective community-based management practices that include the establishment of marine reserves. However, we also show that CBM can collapse when local communities lack linkages to higher levels of governance that help legitimize their organizational efforts [14]. We illustrate the potential for rapid rise and fall of communal self-governance in young fisheries through an effort for CBM of a network of marine reserves in Northwest Mexico.

**Figure 1:**
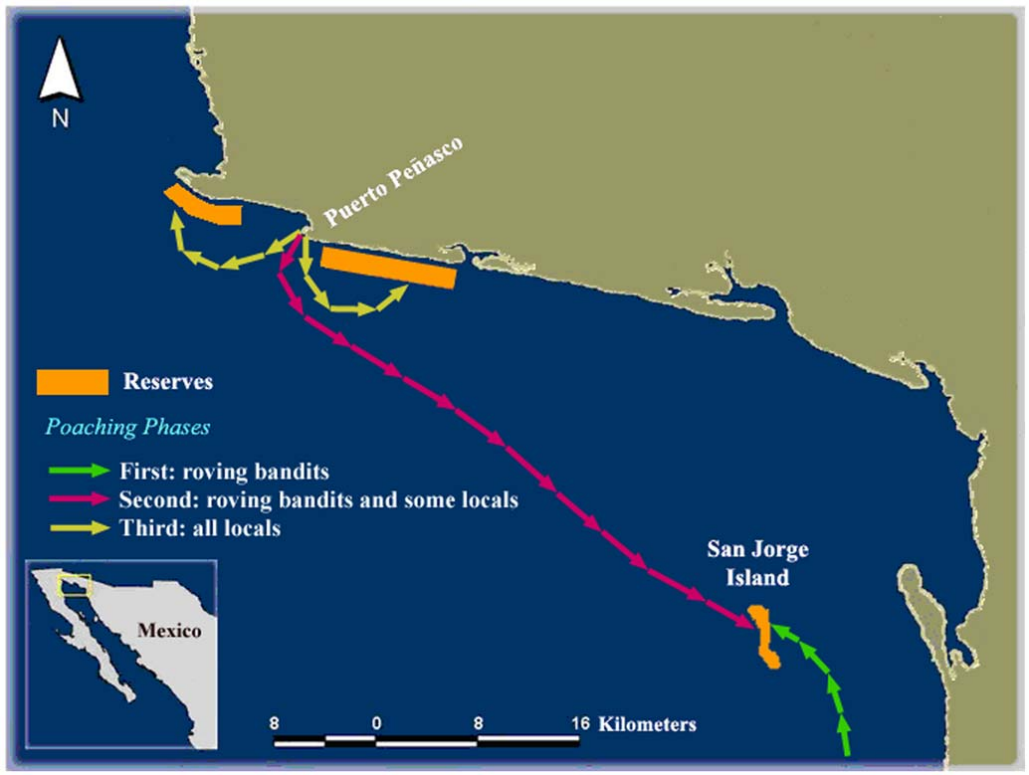
Community-based marine reserve network of Puerto Peñasco and sequential phases of poaching. During the first phase (green), divers from other locations, “roving bandits”, poached on the San Jorge Island reserve. This was followed by local rule breaking and some divers from Puerto Peñasco poaching on the Island (second phase, pink arrows). During the third phase (yellow), all members of the Puerto Peñasco diving cooperative broke their local rules and coastal reserves were targeted. It took less than two months for rules to be broken by all local divers after entrance of roving bandits.

Based on extensive ecological and social studies conducted prior, during, and after reserve establishment, we observed the evolution of CBM efforts in a recently organized inshore fishery of the Gulf of California, Mexico. These efforts - which included the establishment of a marine reserve network and locally enforced harvesting rules - led to a substantial and documented increase in local resource abundance [15]. The network includes an offshore reserve surrounding an island and two coastal reserves, providing protection to roughly 30% of a fishing sector's fishing grounds (Fig. 1). Reserves were created by a cooperative of 22 commercial divers of Puerto Peñasco, a fishing and tourism hub located in the northeastern portion of the Gulf of California, as a means to protect and enhance mollusk stocks, particularly rock scallops (*Spondylus calcifer*) and black murex snails (*Hexaplex nigritus*), two staple resources of commercial divers [16]–[18]. The divers' cooperative has been harvesting benthic shellfish from rocky reefs and adjacent sandy areas for approximately 30 years [19]. The cooperative is comprised of no more than two generations of divers (age range 24–58 yrs) who arrived from communities south of Puerto Peñasco in the late 1970's [19]. While the fishing cooperative in its current structure was formally established in 2001, some members belonged to a similar cooperative before that and had been harvesting benthic resources since the late 1970s. Some of these older fishers were leaders in the new cooperative and played an important role in creating management measures.

Local divers created and enforced the reserves while working closely with researchers from a local non-governmental conservation organization (Centro Intercultural de Estudios de Desiertos y Océanos) and an academic institution (the University of Arizona) to design and implement a monitoring program for their fisheries (Cudney-Bueno worked with these institutions and the cooperative of divers between 1997–2004, helping facilitate fishers' organizational meetings and leading the monitoring program with commercial divers). Fishers approached these institutions in 1998 to help conduct population assessments and biological studies of their fisheries. Close collaboration between fishers, researchers, and local institutions emerged, leading to the subsequent design, establishment, and enforcement of the network of marine reserves four years after this collaboration began [19]. The network was designed by fishers based on their knowledge of local currents, of differences in densities of mollusks, and recollection of previous abundant sites that the cooperative wanted to see rebound. Researchers worked with fishers to help bridge their local knowledge with experimental design and establish a monitoring program to measure changes within and outside the reserves [19]. Reserve establishment occurred without waiting for official government recognition at a time when other efforts for the establishment and management of marine reserves in the region had been highly conflictive [20].

Undoubtedly, the presence of researchers and an NGO played an important role in facilitating the establishment of reserves. However, we do not elaborate on the conditions or the relative importance of factors that led to the establishment of reserves since this is discussed elsewhere [19] and is forthcoming work. Instead, we describe the main components of the CBM system, its effects on local resources and cooperation, as well as the processes and factors leading to a downfall in local governance and subsequent effects on resources protected. We discuss these results in the context of the “tragedy of the commons” paradigm and emerging worldwide efforts to foster CBM of fisheries.

## Results

### Local Management of the Commons

Community-based management of the marine reserve network relied primarily on a suite of simple rules and means of enforcement, leadership of key individuals, meeting venues that allowed for social and ecological feedbacks, and capitalizing on the region's physical and environmental characteristics.

#### Formal and Informal Rules and Sanctions

Fishers designed, monitored, and enforced three main forms of rules: resource-based rules (snail fishing banned June and July; fishing banned within reserves), monitoring rules (mandatory participation in and financial contributions for monitoring) and administrative rules (mandatory: participation in cooperative meetings, monthly financial contributions to the cooperative, and timely provision of paperwork for cooperative). These rules and their sanctions were built primarily on foundations of trust and reciprocity and concerns for the group's well being. Hence, the most effective and usual form of enforcement relied on variations of peer pressure and public shame. This, in essence, could ultimately threaten the rule-breaker's reputation and his social bonds and norms—also known as social capital [21]—with the rest of the members of the group. These were *de facto* sanctions with no legal standing under the statutes of the cooperative. While other formal sanction types were developed, they were either largely avoided, often changed, or were applied last (Table 1). On occasions, local government officials provided enforcement support that was based entirely on the rapport built between fishers and officials, as reserves were yet to be formalized by the government.

**Table 1:** Formal and informal sanctions by rule type devised by local fishers.

Rule Type	Sanction Types	Formal (F)/Informal (I)
**Resource-based rules**	On site warnings and verbal confrontations	I
	Peer pressure/public shame during meetings	I
	Threats of temporary confiscation of boat	F
	Threats of expulsion from the cooperative	F
**Monitoring rules**	Peer pressure/public shame during meetings	I
	Extend rule breaker's monitoring responsibilities	F
**Administrative rules**	Peer pressure/public shame during meetings	I
	Threat of expulsion from cooperative after 3 faults	F
	Temporary confiscation of boat	F
	Expulsion from the cooperative	F

To exemplify this, 100% of fishers interviewed said that they trusted that other fellow fishers for the most part respected the reserves. Similarly, when asked the open ended question “In what way would breaking cooperative rules affect you?” all answers fell into three categories: 1) personal guilt and sense of betrayal to the group, 2) concern of the rest of the group's opinion about one's actions, and 3) concern over the possibility of losing trust and friendship.

If someone within the group cheated, the first approach of members of the cooperative was to tap into the personal guilt associated with the event. Often, it was only necessary to bring the case to the attention of the group without singling out specific people. “Cheaters” assumed that at least someone else likely knew who the culprit was. This way, the informer's reputation was also protected and he would not be labeled as an accuser. “Accuser” is one of the worst labels a commercial diver can have, largely because it can undermine his network ties and reliance on these ties when in need of any help. During interviews, when given a choice to express what would be worse, for the group to label you as an accuser or as a cheater, practically all divers found it impossible to make a choice. They were both seen as equally detrimental.

#### Role of Leadership and Cooperative Meetings

When direct accusations in front of the group did take place, however, these were carried out by the more elder or experienced divers who had already gained high levels of respect within the group. These individuals played a pivotal role during meetings. They gave credibility to agreements and helped maintain, although often contentious, a respectful meeting atmosphere. They were also the main players involved in confronting cheaters directly on site when found breaking any given rule.

Cooperative meetings encompassed a key component for the evolution of cooperation and maintenance of checks and balances. Between summer 2002 and summer 2004, 15 meetings were held, all with an attendance of at least 80% of members. More than acting as a means to discuss various issues pertaining to administration, these meetings provided the main venue to maintain the checks and balances of the system and its functionality. They provided a forum for the development of trust, the generation of rules and sanctions, and allowed for collective feedbacks from biological knowledge gained while commercial diving and/or monitoring. This, in turn, reinforced among the group the perceived benefits of the reserves and played a key role in dismissing poaching allegations and re-enforcing group strength. For instance, it was common for rumors of poaching to develop and quickly spread within the group. However, these rumors were usually dismissed during cooperative meetings. Given that fishers were directly involved in the monitoring process, with designated individuals repeatedly monitoring the same areas jointly with academic researchers, there was a strong sense of individual appropriation towards each monitored site and of other group members' respect towards the reports or opinions of these individuals. Knowing that poaching allegations were often false and that resources were in good health would in turn re-enforce the unity and strength of the group and trust in its members. In short, burden of proof regarding the state of reserves and fishing areas fell largely on fishers themselves.

#### Role of the Physical and Environmental Layout

Knowing if, when, and where a poaching event took place was facilitated by the region's physical and environmental layout. It is simple to know where a diver fishes on a daily basis. The group is small and highly communicative, allowing for the quick spread of information. Coastal reserves are found close to port and fishing activities within them can be easily detected either from shore or from fishing areas. In addition, diving patterns within any given month are constrained by environmental factors, particularly tidal currents and visibility. For instance, during monthly spring tides, when tidal currents are strongest, divers are largely constrained to dive within the reefs south of port. During neap tides, they target offshore areas and the reefs north of port.

In the case of San Jorge Island, which is found farther offshore and is harder to patrol, enforcement relied on more active means. These means, however, were geared towards patrolling entrance of outsiders and not of members of the cooperative. It is quickly known when a diver from Puerto Peñasco goes to the island as this trip demands extra preparation and usually involves overnight stays. Cooperative members would sometimes carry out trips to the island during neap tides with the sole purpose of seeing if anyone was there. However, on three occasions when credible rumors emerged about outsiders poaching, Puerto Peñasco divers also gained the support of the local Navy and fisheries offices to assist in patrolling and enforcement operations. This collaboration was based on the rapport built between divers and local government officials throughout the years rather than as a mandate, as reserves were yet to be formalized at a federal level.

The unique environmental characteristics of the region also facilitated local divers' efforts to discourage settlement of outsiders. A case in point is when a prominent Puerto Peñasco buyer hired divers from another region to work for him at low wages and increase his revenues. Local divers advised them to fish in areas and times marked by intense currents and low visibility. These divers never developed the skills to dive in the region and left soon after.

### Outcomes Strengthen Cooperation

This relatively informal governance system was highly effective. Regular underwater monitoring visits to reserve sites revealed minimal evidence of fishing activity within reserves. Finding evidence of rock scallop fishing is facilitated by the fact that the right valve remains attached to the rock after the scallop muscle has been removed and its bright white color contrasts with the rest of the reef. In the case of black murex snails, these are only harvested when they form large summer breeding aggregation mounds [18]. We saw the same aggregations repeatedly in reserve sites. Similarly, we confirmed only 13 poaching events in at least 2,000 fishing trips conducted between Summer 2002-end of Spring 2004. Hence, rule compliance was high and accomplished primarily through means of social pressures rather than through heavy policing by external officials.

**Figure 2:**
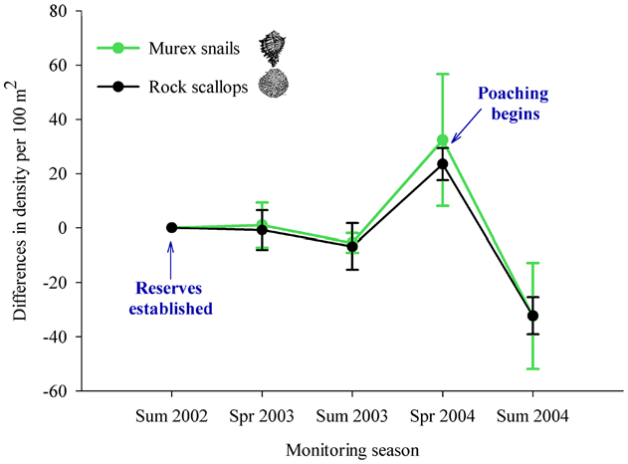
Rapid rise and fall of San Jorge Island fishery stocks from summer 2002, when the Puerto Peñasco community-based reserve network was established, to the end of summer 2004, three months after roving bandits poached on the island. The graph depicts differences in relative densities (S.E. bars included) from one monitoring season to another for the main species harvested: murex snails (*Hexaplex nigritus*) and rock scallops (*Spondylus calcifer*).

Only two years after the establishment of the reserve network, populations of black murex and rock scallops had increased markedly on the San Jorge Island reserve (Fig. 2) [also see 15], with relative densities of up to 160 individuals per 100 m^2^ that exceed any others reported for the Gulf of California [22], [23]. As has been shown in other cases [24], positive effects were also seen in fishing areas adjacent to the reserves [15]. Relative densities of juvenile rock scallop had increased by up to 40.7% within coastal reserves and by 20.6% in fished areas, and changes were also evident for black murex, with more than a three-fold increase in density of juveniles within fished areas [15]. This increase in density of juveniles, combined with predictions from larval transport models and field oceanography data, indicate enhanced recruitment via protection of larval sources within the reserve network [15].

**Figure 3:**
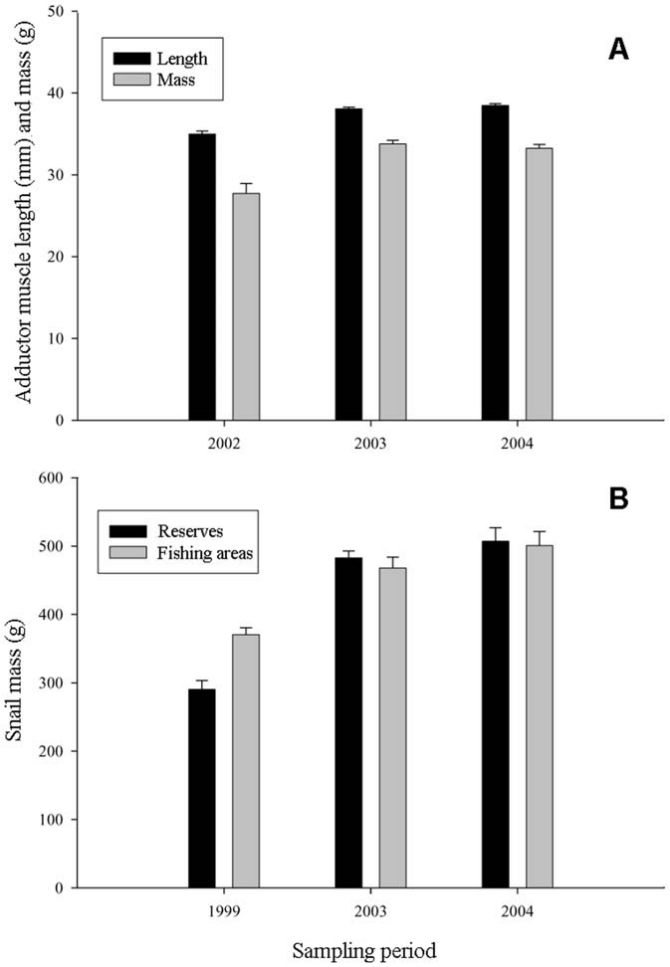
Changes in length and mass of rock scallops (*Spondylus calcifer*) and black murex snails (*Hexaplex nigritus*) before and after reserve establishment. (a) Comparison of the average adductor muscle length and mass of rock scallops from fishing areas (2002 = Spring, two months before reserve establishment). Data from reserves was not obtained as animals would have needed to be sacrificed. (b) Comparison of the average live mass of black murex snails from reserve and fishing areas.

Data from divers' catches of rock scallop showed an increase in average mass of 19.9% (*F*_2, 897_ = 10.78; *p*<0.0001, 1-way ANOVA) in the two years since reserve establishment (Fig. 3A). Similarly, average mass of black murex increased by 74.74% in reserves (*F*_2, 220_ = 77.75; *p*<0.001, 1-way ANOVA) and by 35% in fishing areas (*F*_2, 421_ = 23.80; *p*<0.001, 1-way ANOVA) (Fig. 3B).

Rapid feedback from fishing resources allowed fishers to expect future benefits of the group's various initiatives. In interviews conducted prior to providing results on monitoring efforts, over 85% of fishers reported benefits from the reserves, wanting to continue the reserves into the future.

### The Fall of Cooperation

Shortly after these initial positive outcomes, local governance faced severe external challenges in the form of: 1) lack of recognition at higher levels of government of the local arrangements, 2) abrupt changes in local government leadership, such as the replacement of the chief of the fishing agency office in Puerto Peñasco, who was a local himself, and (3) new fishing pressure from outsiders. News about these community-based management efforts and the abundance of resources at the reserves spread quickly at a regional scale. “Roving bandits” from more than 300 km away (along the coastline, eight hours travel by boat) began fishing the island. Since reserves and territorial use rights were not formally recognized by the Government, local fishers did not have the right to expel others from their reserves and *de facto* fishing grounds. Doing so could put them and the cooperative at risk. Furthermore, they no longer had any support of local government officials, which reduced even more any options available to cope with the problem. Various cooperative members confronted outsiders verbally on at least 3 different occasions and physical confrontation was seriously considered, an option that was soon dropped as it would have had serious legal consequences on the cooperative and those involved. Unable to deter poaching, those local divers who historically had fished on the island the most opted to fish there before outsiders finished reaping the benefits of their own investment. What followed was a rapid cascading effect on fishing resources and locally-designed rule compliance. The island quickly became a free for all. In one month, mollusk populations were reduced in half (Fig. 2). As the island was being harvested by most cooperative members, local divers who tend to fish closer to shore also began to target coastal reserves even without direct pressure from roving bandits in these areas. There were no incentives to continue protecting coastal reserves knowing that fellow fishers were obtaining large catches offshore and that prices would likely fall due to market saturation. Coastal reserves also became an opportunity to rapidly harvest in a setting with limited competition from other fellow fishers. In less than two months since roving bandits first entered the area, all reserves had been targeted by every member of the cooperative at least once (Fig. 2 and Table 2).

**Table 2:** Rules and levels of compliance before and after entrance of roving bandits†.

Rule Type	Compliance Time A (Before)	Compliance Time B (After)
**Resource-based rules**		
Snail fishing banned May-July	1	5
Fishing banned within reserve network	1	5
**Monitoring rules**		
Participation in monitoring	1	5
Financial support for monitoring	1	5
**Administrative rules**		
Participation in all meetings	1	4
Monthly financial contribution	2	5
Providing paperwork necessary for the cooperative	2	2

† Compliance levels based on percentage of fishers known to have broken the rule at least once: 1 = very low (<10%), 2 = low (10–40%), 3 = moderate (41–60%), 4 = high (61–90%), 5 = very high (>90%). Time A = June 2001–May 2004, Time B = first six months (June–November 2004) after entrance of roving bandits.

Social capital that initially allowed for a rapid evolution of cooperation and self-governance for resource management now facilitated overexploitation and rule breaking (Table 2). Once most members of the cooperative had broken some rule, accountability had been eroded and fishers were no longer willing to hold their peers accountable for poaching due to strong social ties with them in other dimensions outside of fishing. Key cooperative members, for instance, stopped attending meetings in order to avoid encounters with specific people, at times family related. Between June 2004 and December 2005, of six cooperative meetings held, only one had the minimum quorum necessary to make decisions recognized under the bylaws of the cooperative (50%+1 members).

## Discussion

A combination and interaction of three main factors led to the initial downfall of cooperation within this CBM system: lack of government recognition, changes in local government leadership, and entrance of roving bandits. Once rule-breaking had become prevalent, these factors were further exacerbated by the existing strong social ties among cooperative members, ties that under other conditions had been conducive to CBM. Although we cannot quantify which factor weighed more over the other, not having formal government recognition of management guidelines and of fishers' *de facto* fishing grounds clearly hampered any means to cope with roving bandits, clouding any possible incentive to cooperate. The importance of this recognition in helping to foster local cooperation is further exemplified with recent developments. In summer 2006, the Mexican Government granted a fishing concession to the Puerto Peñasco diving cooperative, providing its members exclusive access rights to fish rock scallop within what were before *de facto* fishing grounds [19]. Rights to the concession demand adherence to a government recognized management plan, which includes the establishment of seasonal and area closures, as well as total allowable catches [19]. Divers are once again conducting subtidal monitoring, are active participants in stock assessments to define their annual quota, have defined new rules for overall governance of the cooperative, and meetings are regularly held at full quorum [personal communication, Iván Martínez, Centro Intercultural de Estudios de Desiertos y Océanos].

The downfall of this CBM effort could be simplistically attributed to a local tragedy of the commons [13]. Realities and outcomes of commons dilemmas can, however, be much more complex and scale-dependent. We have shown that cooperation for community-based management of a local commons, in which marine reserves form a core component of the system, can indeed emerge and evolve rapidly in a fishery with limited collaborative experience. Locals can find an effective set of rules to self-govern their marine resources and with which sustainable resource exploitation is more likely to take place. This cooperation in turn can be reinforced by perceived rapid positive outcomes of local management interventions. However, as effective a CBM system may be at a local scale, it is likely that this effectiveness may only last as long as the system remains buffered from external pressures.

It is important that CBM efforts that incorporate marine reserves are initially implemented in systems where responses can be measured rapidly and where there is an existing social base for reserve establishment. Reserves reduce the total fishing area, initially render an economic cost to fishers, and complicate management of risk by reducing the physical spaces available to choose from in accordance to variations in environmental conditions and the state of their resources. Stakeholder participation in monitoring, where there is a rapid feedback of the system's response, can play a key role in reinforcing cooperation. In this regard, some sessile or semi-sessile fisheries with rapid growth rates can be good candidates to invest and promote the emergence of social capital and reserve establishment. They can provide a fast feedback to fishers and a setting that can be tinkered with [19], [25], eventually leading to developing some of the factors associated with ways of organizing activities that are sustainable for the long term [26].

Nevertheless, even if CBM efforts are effective within the local biophysical and social context, we show that cooperation and strong social capital alone are not enough to sustain their efficacy. Fishers and fishing communities need to be granted formal government recognition of their locally-devised management structures when they appear to be effective. Higher levels of governance have the ability to create incentives for the emergence of local cooperation leading to sustainable resource use. One way of providing incentives for successful CBM is by rewarding such efforts with formal cross-scale governance recognition and support.

In an increasingly globalized economy, the existence of isolated and buffered fishing communities has largely been lost. Yet, as we show, effective CBM that includes costly decisions like the establishment of marine reserves can emerge even in these settings. Not granting appropriate forms of territorial use rights nor formally recognizing and giving viability to effective local management structures and arrangements, as simple or complex as these may be, could threaten a community's existing foundations for sustainable use of fishery resources. In short, without effective cross-scale institutional arrangements in place that provide robustness to a CBM system, just as cooperative behavior can arise it can also fall along with the biological resource base intended to be managed.

## Materials and Methods

Our research followed a mixed method approach that combined qualitative and quantitative research in the social and biophysical sciences, including the development of larval dispersal models to assess reserve effects within and outside of reserves. Comprehensive results and analyses of the biophysical research are provided elsewhere [15]. At the broadest level, our findings build on social and ecological research with commercial divers of Puerto Peñasco (conducted by Cudney-Bueno) before (1997–2001), during (2002) and after (2003–2004) the establishment of marine reserves. This timeframe entails more than 600 days living in Puerto Peñasco, participation in 147 commercial diving trips, and attending 30 meetings of the *Sociedad Cooperativa Buzos de Puerto Punta Peñasco*, Puerto Peñasco's divers' fishing cooperative.

Research was based on principles of participatory research [27], where stakeholders are actively involved in research and decision-making. Fishers formed part of the research process by having designed and established their marine reserves and monitored the state of their fishery resources within these. Before monitoring began, commercial divers were trained by R. Cudney-Bueno. All biological monitoring was conducted by academic researchers in collaboration with divers.

Following is a summary of the methods used to address the effects and evolution of the Puerto Peñasco community-based marine reserve initiative.

### Social Qualitative and Quantitative Research

Ethnographic research on the Puerto Peñasco diving fishery began five years before the establishment of marine reserves, which allowed us to address social dynamics prior to and after the establishment of reserves. Between Summer 2003–2004, Cudney-Bueno conducted fieldwork specifically targeted to address a) if current collective action for the establishment of marine reserves developed quickly and with no or very limited previous experience to define and/or establish collective management decisions, and b) the conditions that facilitated and led to the establishment of community-based management efforts. Through oral histories, we searched for previous cooperative efforts and key past events or situations that could have shaped fishers' interests in adopting more conservation-oriented measures. Oral histories also allowed us to single out and understand relevant issues that may not be as clearly or obviously identified with the use of directed questions. Full results and analyses of this ethnographic research go beyond the scope of this paper and are in preparation.

We complemented our qualitative research with structured interviews. Throughout March and at the beginning of April 2004, we conducted structured interviews with 18 fishers, representing 82% of the members of the diving cooperative of Peñasco. These interviews primarily addressed perceptions of fishers as to the effects and efficacy of their management efforts, factors affecting the evolution of cooperation within the cooperative such as the building of trust among cooperative members. We conducted all interviews at fishers' homes.

Having had the time to build sufficient rapport and trust with local fishers, it became possible to gain a comprehensive understanding of the diving fishery, how divers define and enforce rules and regulations, record the presence or absence of poaching events, and note if conflict resolution and consensus-building processes were facilitated or halted.

### Quantitative Estimation of Population Parameters

We estimated changes in relative densities of rock scallop (*Spondylus calcifer*) and black murex snail (*Hexaplex nigritus*) in reserve and fishing sites for two consecutive years beginning in Summer 2002, one month preceding reserve establishment. These species were selected for being the main species targeted by the commercial diving fishery and representing the main reason leading to the establishment of the reserves.

The region monitored encompassed the reefs of San Jorge Island and those found near the fishing town of Puerto Peñasco (within 3 km from highest tide line) in the eastern part of the northern Gulf of California, Mexico. This region extends from 31,22,18.1 N; 113,39,09.4 W to 31,15,03.8 N; 113,20,48.1 W (see Fig. 1).

We subdivided the region into 5 sampling areas: a) two coastal reserves, Las Conchas and Sandy; b) two coastal fishing areas, Los Tanques and La Cholla; c) one offshore island reserve, San Jorge Island (Fig. 1). We conducted density counts of juveniles and adults in 58 100 m^2^ permanent quadrants distributed randomly across these 5 sampling areas. To reduce heterogeneity associated with depth, we restricted all sampling to depths ranging from 40–65 ft. This also reduced health risks associated with diving and facilitated overall monitoring as we were able to remain underwater for longer periods of time. A comprehensive account of methods used to analyze reserve effects on recruitment within and outside reserves - which combines results from monitoring data, outputs of larval transport models, and field oceanography - is provided elsewhere [15].

### Quantitative Estimation of Changes in Size and Mass of Harvested Species

We estimated changes in the size and mass of adult (harvested) black murex and rock scallop. For black murex, we collected specimens (n = 244) from breeding aggregations of reserve and fishing sites before the establishment of reserves (Summer 1999) and after their establishment (Summer 2003 and 2004). All snails from reserves were returned to the collecting site, whereas snails from fishing sites were obtained from fishers' catches [for details on sampling black murex see 16].

For rock scallops, we estimated changes in average length and mass of the adductor muscle, the part of the animal that is commercialized and that fishers return to port. Since the only way of obtaining samples of the adductor muscle is by killing the animal, we limited our samples and analyses to fishing sites. All samples were constricted to Spring (post reproduction) to avoid variations in weight and size caused by glycogen accumulation in the muscle pre and post reproduction [17]. We obtained a total of 1081 samples of rock scallop from Spring 2002 (pre-reserve establishment) to 2004 [see sampling details in 17]. Both black murex and rock scallop data were analyzed using a 1-way ANOVA framework and Tukey's HSD multiple comparison test to determine pairwise relationships.
